# Low-Copy Genes in Terpenoid Metabolism: The Evolution and Expression of *MVK* and *DXR* Genes in Angiosperms

**DOI:** 10.3390/plants9040525

**Published:** 2020-04-19

**Authors:** Natacha Silva, Suzana Tiemi Ivamoto-Suzuki, Paula Oliveira Camargo, Raíssa Scalzoni Rosa, Luiz Filipe Protasio Pereira, Douglas Silva Domingues

**Affiliations:** 1 Departamento de Biodiversidade, Instituto de Biociências, Universidade Estadual Paulista, UNESP, 13506-900 Rio Claro-SP, Brazilsuzanatiemi@yahoo.com.br (S.T.I.-S.);; 2 Laboratório de Biotecnologia Vegetal, Empresa Brasileira de Pesquisa Agropecuária (Embrapa-Café), 86047-902 Londrina-PR, Brazil; filipe.pereira@embrapa.br

**Keywords:** *Coffea*, MVA and MEP pathways, purifying selection, RT-qPCR

## Abstract

Terpenoids are a diverse class of metabolites that impact plant metabolism in response to environmental cues. They are synthesized either via a predominantly cytosolic (MVA) pathway or a plastidic pathway (MEP). In *Arabidopsis*, several enzymes from the MVA and MEP pathways are encoded by gene families, excluding *MVK* and *DXR*, which are single-copy genes. In this study, we assess the diversity, evolution and expression of *DXR* and *MVK* genes in selected angiosperms and *Coffea arabica* in particular. Evolutionary analysis revealed that *DXR* and *MVK* underwent purifying selection, but the selection effect for *DXR* was stronger than it was fo*r MVK*. Digital gene expression (DGE) profile analysis of six species revealed that expression levels of *MVK* in flowers and roots were high, whereas for *DXR* peak values were observed in leaves. In *C. arabica*, both genes were highly expressed in flowers, and *CaDXR* was upregulated in response to methyl jasmonate. *C. arabica* DGE data were validated by assessing gene expression in selected organs, and by plants treated with hexanoic acid (Hx) using RT-qPCR. *MVK* expression was upregulated in roots treated with Hx. *CaDXR* was downregulated in leaves by Hx treatment in a genotype-specific manner, indicating a differential response to priming.

## 1. Introduction

Terpenoids are a large and diverse class of metabolites that include compounds essential for cellular functions and environment interactions [1]. They are the largest and most diverse class of metabolites, containing over 40,000 substances. The molecules are industrially relevant and are used as flavors, pigments, polymers and drugs [1,2].

Terpenoids are produced in all living organisms, but they are most abundant and possess a greater degree of diversity in the plant kingdom [1]. Their biological functions affect plant membrane structure (sterols), respiration (ubiquinone), photosynthesis (chlorophylls, carotenoids, prenylquinones) and the regulation of plant development (cytokines, brassinosteroids, gibberellins, abscisic acid, strigolactones) [3].

They are derived from two precursor molecules: isopentenyl diphosphate (IPP) and its isomer, dimethylalyl diphosphate (DMAPP) [4]. There are two pathways used to produce IPP and DMAPP in higher plants: the mevalonate pathway (MVA) and methylerythritol phosphate (MEP) pathways. The MVA pathway is predominantly in the cytosol but is also distributed between the endoplasmic reticulum and peroxisomes [5,6], and the MEP pathway is located in plastids [7]. The key rate-limiting enzymes of the MEP pathway and the MVA pathway are, respectively, the extensively studied 1-deoxy-D-xylulose-5-phosphate synthase (DXS) and hydroxymethylglutaryl-CoA reductase (HMGR) [7,8,9,10]. However, there are enzymes in the MVA and MEP pathways that are still poorly addressed.

The *mevalonate kinase* (*MVK*) gene encodes one important biosynthetic enzyme in the MVA pathway [3]. It catalyzes the phosphorylation of mevalonate to produce 5-phosphate mevalonate [3]. *MVK* activity is involved in plant regeneration and growth, and it is also related to the frequency of shoot and root formation in white pine (reviewed in [7]).

1-deoxy-d-xylulose 5-phosphate reductoisomerase (DXR) is also an important enzyme involved in terpenoid biosynthesis via the MEP plastid pathway, and is responsible for catalyzing the second step of terpenoid biosynthesis in the chloroplast [1]. Inactivation of the *1-deoxy-D-xylulose-5-phosphate reductoisomerase* (*DXR*) gene results in strong developmental arrest and albino plants in *Arabidopsis* [11].

Only plants are capable of simultaneously synthesizing terpenoids via MEP and MVA pathways in parallel, suggesting that genes involved in terpenoid biosynthesis in plants have adapted evolutionarily in basal to terrestrial plant lineages [12,13]. In this sense, it is important to understand the evolutionary relationships and duplication events that have occurred within these gene families [3]. Gene duplication, mutation and natural selection are essential processes that expand plant genetic diversity and facilitate biological adaptation [14,15]. Phylogenetic studies can provide important information needed to enhance our knowledge regarding the mechanism by which *MVK* and *DXR* genes influence terpenoid biosynthesis in higher plants. These genes are both single copy genes in the model plant *Arabidopsis thaliana*. Although previous studies report the phylogenetic analyses of *MVK* and *DXR* [16,17,18], these studies were focused in species-specific gene characterization and did not seek to further investigate the evolutionary aspects of *MVK* and *DXR* in plants.

The *Coffea* genus (Rubiaceae) is comprised of 124 species [19]. *Coffea arabica* and *C. canephora* are of the species of greatest economic importance and have garnered worldwide interest [20]. Few molecular studies have addressed the terpenoid biosynthesis pathways in coffee plants. Previously, researchers investigated the roles of the *3-hydroxy-3-methylglutaryl-CoA reductase* (*HMGR*) gene, which is responsible for the third step of the MVA pathway, and their respective expression profiles in several organs [21]. Another study validated the function of three *C. arabica* monoterpene synthases (limonene, linalool and β-myrcene synthases), and studied the evolution of the genes, which affect the composition of coffee aroma [22]. Thus far, there have been no studies addressing the molecular responses of hub genes of these pathways.

Resistance inducers are known to modulate the expression pattern of genes involved in terpenoid synthesis [23]. Hexanoic acid (Hx) is a short, naturally occurring monocarboxylic acid that induces resistance to pathogens in several plant systems [24]. In *Citrus* plants, Hx has been shown to induce genes involved in the MVA pathway [25]. This prompted us to hypothesize that terpenoid pathway genes can be modulated by the application of Hx in coffee plants.

The main goal of this study was to enhance our understanding of the evolution of both *DXR* and *MVK* genes in angiosperm species, and to characterize the transcriptional profiles of these genes in *C. arabica* plants. Our results provide important knowledge regarding the evolutionary dynamics of the *DXR* and *MVK* gene families, and evaluate differences in expression via assessment of gene transcription. In addition, these genes can be further analyzed to verify whether they are involved in plant defense mechanisms aimed at enhancing the biosynthesis of terpenoids.

## 2. Results

### 2.1. Copy Number of MVK and DXR Genes in Plants

**Figure 1:**
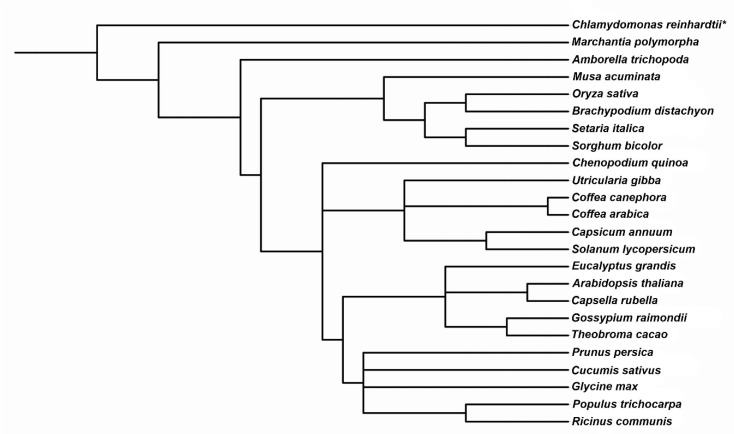
Phylogenetic tree of the analyzed species based on NCBI species taxonomy. This tree was constructed with PhyloT [26] and edited using iTOL [27]. An asterisk (*) indicates that the species lacks an *MVK* gene.

We used a diverse array of angiosperm species to perform genome-wide analysis of *MVK* and *DXR* genes and three species as outgroups: one basal angiosperm, one basal Viridiplantae and one Chlorophyta (Figure 1). Thirty-one *MVK* and 31 *DXR* sequences were identified in 24 species. We identified 22 *MVK* and 21 *DXR* genes in 16 eudicotyledonous species, while in monocotyledons we identified 7 *MVK* and 6 *DXR* sequences in five species. In the three outgroup species assessed, we identified 2 *MVK* and 4 *DXR* genes, except in the unicellular green algae, *Chlamydomonas reinhardtii,* which does not have a gene encoding *MVK* (Table 1). *Chenopodium quinoa*, *Glycine max*, *Gossypium raimondii*, *Marchantia polymorpha*, *Populus trichocarpa*, *Ricinus communis*, *Musa acuminata* and *Setaria italica* had between two and three genes encoding *MVK* and *DXR* (Table 1). As expected, predictions of the subcellular localization of MVK and DXR proteins for all 24 species revealed that MVK is likely located in the cytosol and DXR is likely located in chloroplasts.

### 2.2. Phylogenetic Analyses

**Figure 2:**
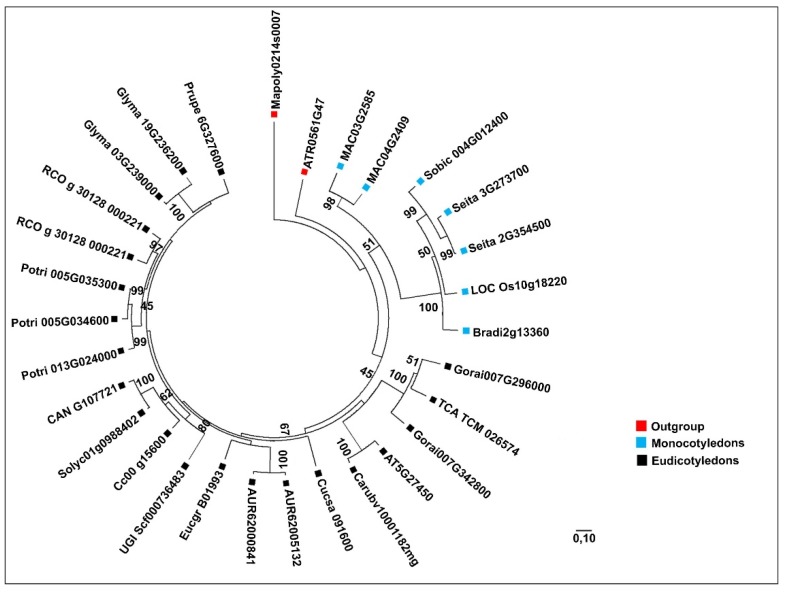
Phylogenetic tree of *MVK* genes from 23 plant species analyzed in this study. Outgroup species genes are indicated in red, while monocotyledon and eudicotyledon genes are indicated in blue and black, respectively. Evolutionary history was inferred using 30 *MVK* amino acid sequences. The tree was made using the maximum likelihood method, based on the LG model, with 1000 bootstraps (shown as percentages). All positions containing missing data and gaps were deleted using Gblocks. There were a total of 360 positions in the final dataset. The evolutionary analysis and tree design were performed using MEGAX software.

**Figure 3:**
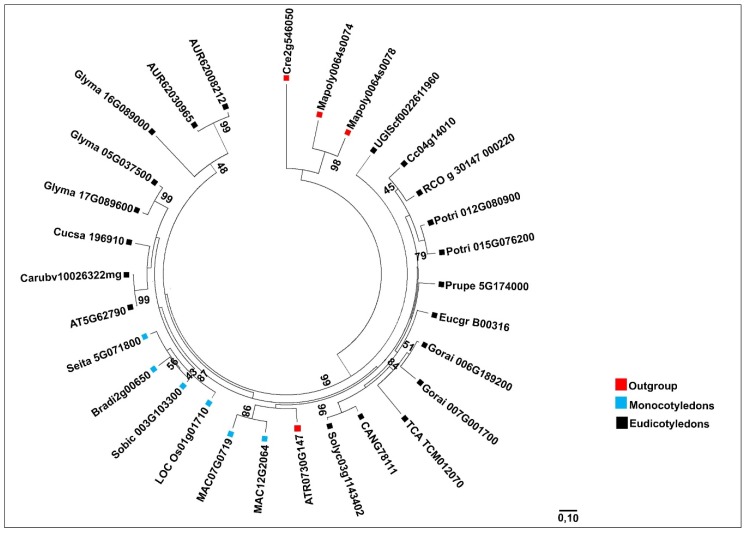
Phylogenetic tree of *DXR* genes from 24 plant species considered in this study. Outgroup species genes, monocotyledon genes and eudicotyledon genes are indicated using red, blue and black boxes, respectively. Evolutionary history was inferred using 30 *DXR* amino acid sequences. The tree was drawn using the maximum likelihood method, based on the LG model, with 1000 bootstraps (shown as a percentage). All positions containing missing data and gaps were deleted using Gblocks. There were a total of 374 positions in the final dataset. The evolutionary analysis and tree design were performed using MEGAX software.

**Figure 4:**
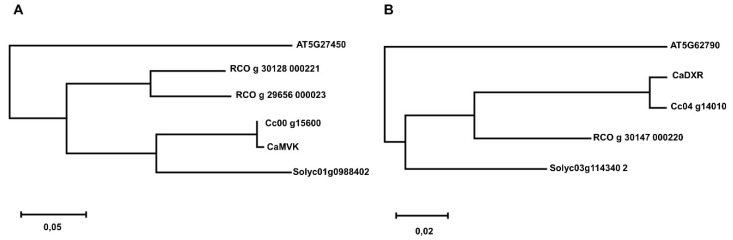
Phylogenetic analysis of *MVK* and *DXR C. arabica* transcripts using five selected species (*A. thaliana, C. arabica, C. canephora, R. communis* and *S. lycopersicum*). The tree was drawn using the maximum likelihood method, which was based on the LG model.

We focused our phylogenetic analysis on plant species whose complete genomes were available within the PLAZA 4.0 database (Figure 2 and Figure 3). The *MVK* gene family was divided into three groups (Figure 2). The first group was composed of outgroup species (red). The second group (blue) contained monocotyledon species and the last group (black) contained eudicotyledon species (Figure 2). The phylogenetic tree of *DXR* revealed a more complex division than *MVK* (Figure 3). We observed one group containing the outgroup species *C. reinhardtii* and *M. polymorpha* (red), while the monocotyledon genes were grouped into one major group (blue) and dicotyledons were divided into two groups (black), one of which was near the outgroup species *A. trichopoda.* In *C. arabica*, *MVK* (comp31934) and *DXR* (comp33448) sequences were retrieved from a transcriptome set previously developed by our group [28]. In this case, we demonstrate that these sequences are closely related to *C. canephora* genes (Figure 4).

### 2.3. Exon–Intron Structure of MVK and DXR Genes

**Figure 5:**
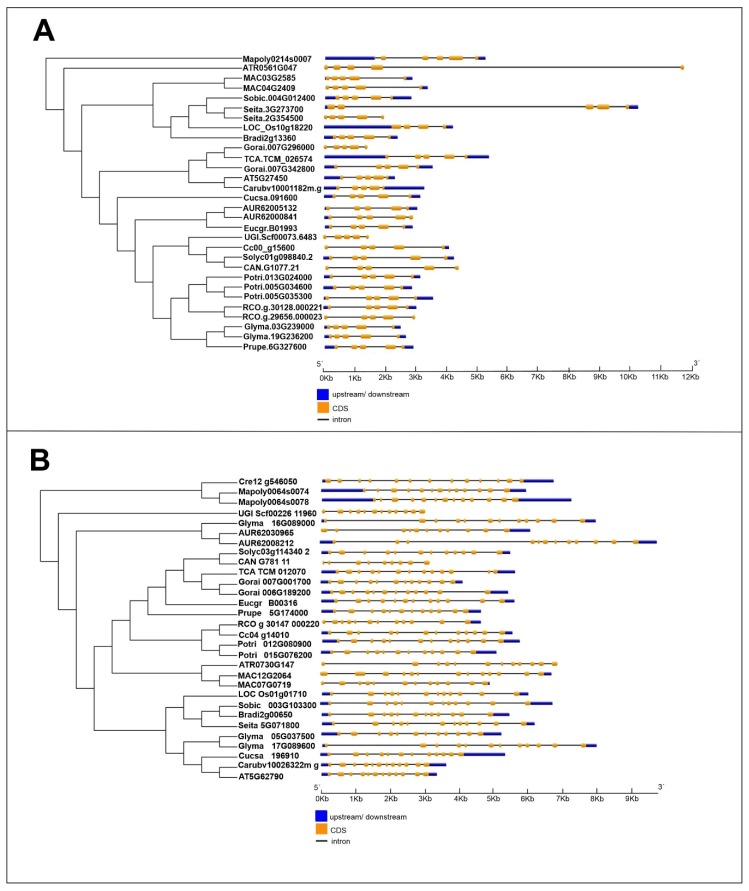
Exon–intron structure of *MVK* (**A**) and *DXR* (**B**) genes.

Exon–intron structures were analyzed to enhance our understanding of the structural diversity of the *MVK* and *DXR* genes of angiosperm species. To perform the analyses, publicly available genomic sequences from the PLAZA database (Figure 5A,B) were used. According to these analyses, the *MVK* gene from all species have five exons, except the *LOC_Os10g18220* gene from *O. sativa,* which has four (Figure 5A). Further, most *DXR* genes have between 11 and 13 exons, but for the *CAN.G1077.21* gene of *C. annuum* we observed only four exons (Figure 5B).

The shortest *MVK* and *DXR* full length gene occur in *U. gibba* (≅ 2Kb), while the longest ~12 kb *MVK* full length gene is from *S. italica* (*Seita.3G273700*) and the longest *DXR* full length gene (*~*10 kb) is in *C. quinoa (*A*UR62008212*) (Figure 5A,B).

### 2.4. Nucleotide Substitution and Selection in MVK and DXR Genes

To verify the possibility of distinct evolutionary pressures, we analyzed the individual selection profile of each amino acid, as well as the non-synonymous (dN) and synonymous (dS) substitutions in eudicotyledonous and monocotyledonous *MVK* and *DXR* genes. Our results showed that the dN/dS ratios of sequences among the *MVK* and *DXR* genes were distinct (Table 2). In addition, all estimated dN/dS values were substantially less than 1, suggesting that all *MVK* and *DXR* sequences within each group assessed are under strong purifying selection pressure. We observed lower dN/dS ratios in *DXR* than *MVK* (Table 2). Both genes had lower dN/dS ratios in eudicotyledons than in monocotyledons, except for *DXR* in model M5 (Table 2).

We found three positively selected sites in eudicots within the *MVK* gene using a Bayesian inference approach to calculate site-specific positive and purifying selection. We also evaluated the identification of sites of positive selection using three methods: SLAC (single likelihood ancestor counting), FEL (fixed-effects likelihood) and MEME (Mixed Effects Model of Evolution). FEL and SLAC software did not detect any positively selected codon sites. MEME analyses, however, did positively identify the selected sites (Table 3). In *MVK*, the MEME model detected three positively selected sites in eudicotyledons and four sites in monocotyledons. Site 42 was identified as a positively selected site in both angiosperm groups. With regard to the analysis of *DXR* using MEME, five positively selected sites in eudicotyledons and two in monocotyledons were identified.

### 2.5. Estimated Time of Duplication of MVK and DXR Genes

The duplication rates of the *MVK* and *DXR* genes were estimated for species with more than one gene copy (*M. polymorpha, G. raimondii, P. trichocarpa, R. communis, M. acuminata, S. italica, C. quinoa* and *G. max*). We used substitution data and inferred nucleotide substitution rates (Table 4 and Table 5). In order to clarify cases of tandem duplication, segmental duplication and whole-genome duplication, we also retrieved a synteny analysis from Plaza database 4.0 (Figure S1).

The *MVK* gene has been duplicated in seven angiosperm species (Table 4). Five species are eudicotyledons (*C. quinoa*, *G. max, G. raimondii, P. trichocarpa* and *R. communis*) and two species are monocotyledons (*M. acuminata* and *S. italica*). In *MVK*, the lowest dN/dS ratio and the most recent duplication event was identified in *P. trichocarpa* (Table 4), which corresponds to a tandem duplication (Figure S1) between two copies of *MVK—Potri.005G034600* and *Potri.005G035300—*which occurred less than 1 million years ago (Figure S1 and Table 5). The oldest event was determined to occur in *M. acuminata* (Table 4). Among the species considered, only *R. communis* and *S. italica* had more than one copy of *MVK* and no recent genomic duplication events were identified [14], suggesting that a species-specific event generated these gene duplications. We identified that *G. raimondii* and *P. trichocarpa* that had undergone a recent whole-genome duplication also have duplicated *MVK* coding genes (Figure S1 and Table 4).

All species with duplicated *MVK* genes also have duplicated *DXR*, except for *S. italica* and *R. comunnis*. Thus, *DXR* duplication appears to be more genome duplication-dependent than *MVK* duplication. *P. trichocarpha* and *G. max* have an unusual profile of the genes in which each have three *DXR* genes and three *MVK* genes, respectively (Table 5).

The synteny analysis (Figure S1) corroborate that most observed duplications correspond to WGD with two cases of local gene duplication. It was not possible to calculate distance for *DXR* in *M. polymorpha* (Table 5).

### 2.6. Recombination Events of MVK and DXR Genes

We identified intragenic recombination events in *MVK* and *DXR* using Geneconv, RDP and MaxChi methods. Five *MVK* genes—in two eudicot species, two monocot species and one outgroup—experienced recombination events (Table 6). A total of eight *DXR* genes—in five eudicot species, two monocots and one outgroup—also had recombination events (Table 7). In the analysis of the *MVK* genes, Seita.3G273700 from *S. italica* had the highest number of recombinations (5). The lowest number (1) was observed in *M. acuminta*, in the gene MAC04G2409 (Table 7). For *DXR*, the highest number of recombinations (6) was observed in *M. polymorpha*, in the Mapoly0064s0074 gene, and the lowest number of recombination events (1) in *T. cacao*, in the TCA.TCM_01200 gene (Table 7).

### 2.7. RNA-seq-Based Expression Profiles

**Figure 6:**
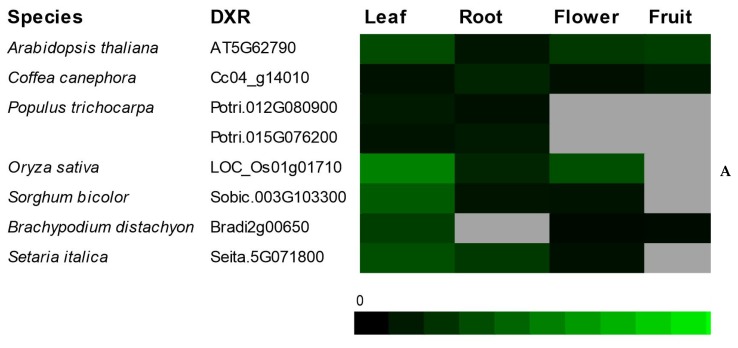

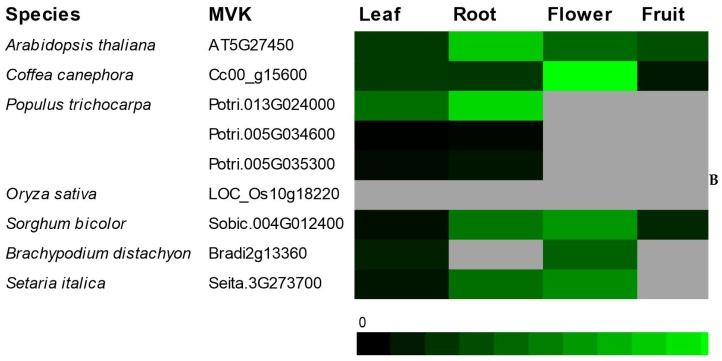
Digital expression profile of *MVK* (**A**) and *DXR* (**B**) genes in organs of *A. thaliana* (AT5G27450 and AT5G62790), *C. canephora* (Cc00_g15600 and Cc04_g14010), *P. trichocarpa* (Potri.013g024000, Potri.005g034600, Potri.005g035300, Potri.012g080900 and Potri.015g076200), *O. sativa* (Os10g18220 and Os01g01710), *S. bicolor* (Sobic.004G012400 and Sobic.003G103300) and *S. italica* (Seita.3G273700 and Seita.5G071800). The black color indicates that there were no transcripts detected, and the gray color indicates that no data were available. The intensity of the green color is proportional to the number of transcripts observed in reads per kilo base per million mapped reads (RPKM) values.

To determine the *MVK* and *DXR* gene expression profiles among angiosperms, we used public RNA-seq data from seven plant species (*A. thaliana, C. canephora, P. trichocarpa, O. sativa, S. bicolor, B. distachyon and S. italica*) and four tissues (leaves, roots, flowers and fruits) (Figure 6). *MVK* transcripts were expressed most highly in *A. thaliana* roots with 56 RPKM (*At5g27450*), *P. trichocarpa* roots with 60 RPKM (*Potri.013g024000*), *C. canephora* flowers (*Cc00_g15600*) with 70 RPKM and *S. bicolor* flowers with 42 RPKM (*Sobic.004g012400*) (Figure 6). *DXR*, on the other hand, was most highly expressed in *A. thaliana* leaves (*At5g62790*) with 181 RPKM *C. canephora* roots with 88 RPKM (*Cc04_g14010*), *O. sativa* leaves with 308 RPKM (*Os01g01710*), *S. bicolor* leaves with 215 RPKM (*Sobic.003g103300*) and *S. lycopersicum* flowers with 608 RPKM (*Solyc03g114340.2*). For both genes, the lowest levels of expression were observed in *P. trichocarpa* in most organs analyzed (Figure 6). Our results indicated that *MVK* is mostly expressed in roots and flowers, and *DXR* in leaves.

**Figure 7:**
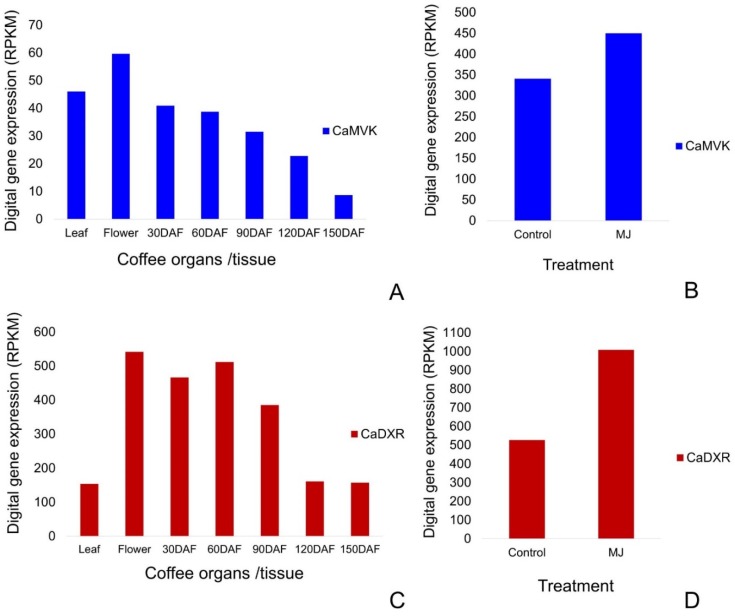
Digital expression profiles of *CaMVK* (blue) and *CaDXR* (red) genes in leaves, flowers and fruits (30 to 150 days after flowering, DAF) in *C. arabica* (**A**,**C**) and under the application of 2mM methyl jasmonate (MJ) (**B**,**D**). Transcript levels are expressed in reads per kilo base per million mapped reads (RPKM) values.

Digital gene expression (DGE) profile analysis of *CaMVK* (Figure 7A,B) and *CaDXR* (Figure 7C,D) from several organs of *C. arabica* was also analyzed. The *CaDXR* (Figure 7C) gene had higher transcript levels than *CaMVK* (Figure 7A) in leaves, flowers and fruits (30 to 150 DAF). However, while *CaMVK* steadily decreases its expression along fruit development, *CaDXS* is decreased only in the late stages of fruit development. In addition, we observed that application of methyl jasmonate upregulated the expression of *CaMVK* and *CaDXR* in fruits (Figure 7B,D).

In order to provide an overall picture of transcriptional responses of MVA and MEP pathways in coffee, we provide DGE analysis of 23 genes of these pathways after MJ application (Figure S2) and in several organs (Figure S3), including *CaMVK* and *CaDXR*. Eleven genes (52.3%) were upregulated similarly to *CaMVK* and *CaDXR* (*CaDXS2*, *CaDXS4*, *CaMCT*, *CaCMK*, *CaHDS1*, *CaHDS2*, *CaIDI*, *CaGGPPS1*, *CaGGPPS2*, *CaAACT2*, *CaHMGS*, *CaMPDC* and *CaIDI*), while six were downregulated (*CaDXS1*, *CaDXS3*, *CaMECPS*, *CaGGPPS3*, *CaAACT1*, *CaPMK*) and two genes (*CaIDS* and *CaHMGR*) did not show significant differences under MJ application (Figure S2). We observed in the fruit development DGE analysis (Figure S3) that 18 genes showed similar expression pattern with *CaMVK* and *CaDXR—*high transcript levels in flowers and low in leaves (*CaDXS2*, C*aDXS4*, *CaCMK*, *CaMECPS*, *CaHDS1*, *CaHDS2*, *CaIDI*, *CaIDS*, *CaGGPPS1*, *CaAACCT1*, *CaAACCT2*, *CaHMGS*, *CaHMGR*, *CaPMK*, *CaMPDC* and *CaIDI*).

### 2.8. RT-qPCR-Based Transcriptional Profile of MVK and DXR Genes in C. arabica

**Figure 8:**
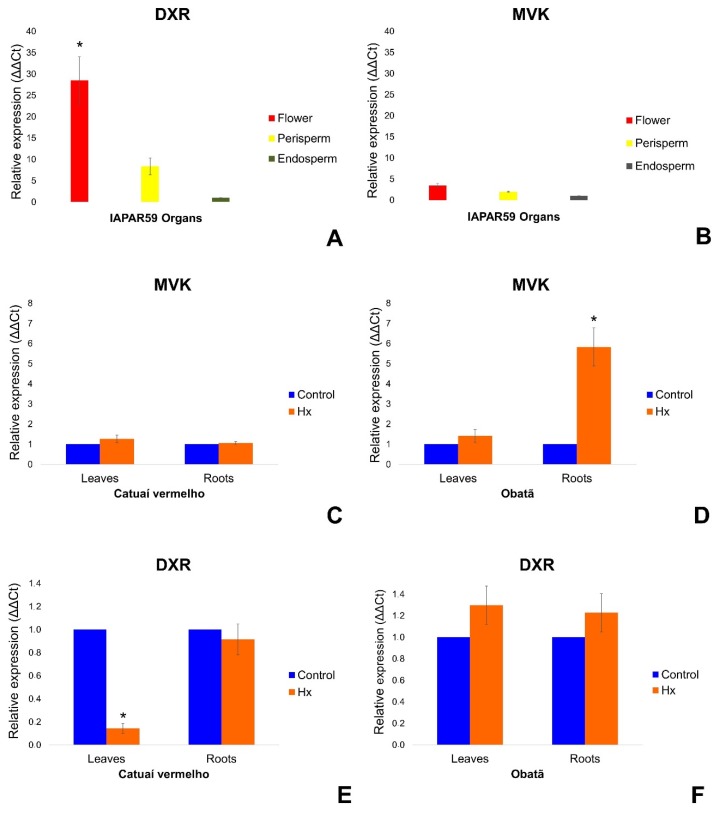
Transcriptional profile of *CaMVK* and *CaDXR* genes in *C. arabica* cv. Iapar 59 (**A**,**B**), *C. arabica* cv. Catuaí Vermelho (**C**,**E**) and *C. arabica* cv. Obatã (**D**,**F**). Experiments were performed using three biological and technical replicates each. Bars indicate standard deviation, and significant differences in which *p* < 0.05 (ANOVA followed by Tukey test) have been indicated with an asterisk (*). Abbreviations: Hexanoic acid application (Hx). Controls represent samples without Hx application.

Levels of *CaMVK* and *CaDXR* gene expression in flowers, endosperm and the perisperm of fruits 120 days after flowering (DAF) were evaluated using RT-qPCR. Both genes were more active in flowers, followed by perisperm and endosperm (Figure 8A,B).

We also verified that exogenous application of hexanoic acid, a well-known natural priming substance, was capable of modulating *CaMVK* and *CaDXR* transcriptional profiles in leaves and roots. *CaMVK* was upregulated in the roots of Obatã cultivar (Figure 8D) post-Hx treatment. *CaDXR* was downregulated in leaves of Catuaí Vermelho (Figure 8E) treated with Hx.

## 3. Discussion

*MVK* and *DXR* are key genes involved in MVA and MEP terpenoid biosynthesis pathways. They produce several terpenoid precursors, however, little is known about their molecular evolution [3]. These genes are present in several plant kingdom lineages, such as algae, mosses, monocotyledons and eudicotyledons [9,10,29]. This study revealed that *MVK* and *DXR* are low copy number genes in all 24 species analyzed. In contrast to *Arabidopsis*, which only has one copy of each gene, several species have more than one copy of *MVK* and *DXR*.

We observed that the outgroup species, *C. reinhardtii,* does not have the *MVK* gene. A previous study has shown that another MVA pathway gene, *HMGR*, was also absent in the species [10]. These data suggest that throughout evolution the MVA terpene pathway may have been lost in Chlorophyta. Unusual patterns of MVA pathway has already been reported in a previous study in bacteria [29].

When analyzing the evolutionary relationships of *MVK* and *DXR* genes among angiosperm species, we observed that the phylogenetic trees displayed distinct clades, which divided outgroup species, monocotyledon and eudicotyledon lineages.

The *MVK* gene tree, clearly divided between monocots and eudicots, is highly similar to *HMGR* [10], suggesting a similar pattern of gene evolution among the MVA pathway. However, *DXR* in eudicotyledons have a reticulate pattern, which was not observed for *MVK*, suggesting differential evolutionary forces acting on the two genes. A reticulated pattern of gene evolution was also observed in *DXS,* the rate-limiting enzyme of the MEP pathway [9], suggesting that this pattern is common for genes related to the MEP pathway.

Previous studies have shown that *DXR* has a greater impact on carotenoid and steroid synthesis than *MVK* [30] and the drastic effect of *DXR* loss on plant development is well known [11]. In this sense, we can also speculate that these observations might be associated with a greater impact of *DXR* on terpenoid metabolism.

Most duplications of these genes were associated with recent WGD (whole-genome duplication) events detected in plant species [15], or to polyploid characteristics of species analyzed. In *C. quinoa*, duplication could be explained by the polyploid character of its genome [31]. In *G. max*, *G. raimondii*, *P. trichocarpa* and *M. acuminata* the identification of more than one copy of *MVK* can be also associated to recent genome duplication events [15]. Interestingly, *R. communis* (Table 4) and *S. italica* (Table 5) possessed more than one copy of *MVK* and did not experience any recent genome duplication events, but increased the number of genes nonetheless. They underwent a possible local gene species-specific duplication of *MVK,* which occurred less than 10 and 5 million years ago, respectively—an unusual occurrence in angiosperms.

Gene structure and organization analyses revealed that the number of introns and exons within these genes are similar among species. However, the *DXR* gene has a more complex exon/intron organization than *MVK*, which may also explain its distinct phylogenetic pattern. This observation is in accordance with our findings, indicating that a greater number of recombination events occurred within the *DXR* gene than the *MVK* gene (Table 6 and Table 7). Gene structure and organization analyses revealed that the number of introns and exons within these genes are similar among species.

We observed that the *MVK* and *DXR* gene families in angiosperms experienced a strong purifying pressure (dN/dS < 1). This fact highlights the functional importance of these genes during their evolutionary process [32]. Some positive sites indicate a possible diversification between strains of both genes in plants [32]. Thus, in sites under positive selection, duplication accelerated, which facilitated functional divergence and resulted in the formation of gene subgroups.

Throughout evolution, gene duplication events may be exposed to divergent selection pressure, which leads to non-functionalization (loss of original functions), neofunctionalization (acquisition of new functions) or sub-functionalization (partition of original functions) [9,10,14]. In this study, dN/dS ratios for duplicated *MVK* and *DXR* genes were less than 1, suggesting that genes were predominantly under strong purifying selection pressure.

To better understand selective pressure, we estimated the age of *MVK* and *DXR* gene duplications using dN/dS data. This confirmed the recent tandem duplication of *MVK* in *P. trichocarpa* and WGD duplications in *G. max*.

We also observed a direct relation between copy number among *MVK* and *DXR* with the respective rate-limiting genes from these pathways—*HMGR* and *DXS*. As examples of gene duplication in *MVK,* we can highlight *G. raimondii*, *G. max* and *P. trichocarpa.* These species were also the ones with higher number of copies of *HMGR* [10]. In *DXR*, *Arabidopsis thaliana* and *Eucalyptus grandis* are species with a single copy of *DXR*; both species have three copies of *DXS* [9]. Conversely, *G. max*, the species with the highest number of DXR copies, also harbored the highest number of *DXS* copies in a similar analysis [9].

Using MEME analysis, we identified amino acid sites that experienced episodic positive selection. We observed an opposite pattern of positively selected sites in *MVK* and *DXR* genes: while more positively selected sites were identified in monocot *MVK* genes, for *DXR* most positively selected sites were in dicot genes (Table 3). The amount of positively selected sites helped us to highlight natural selection effects on individual sites that are usually hindered by the low averaged dN/dS values.The analysis of dN/dS among individual amino acid residues in *DXR* (Figures S4 and S5) also indicated that higher values were in the N-terminal, where the protein has its targeting signal to organelles [33].

The transcriptional profile of genes involved in the initial steps of terpenoid biosynthesis in the *Coffea* genus has, thus far, been poorly investigated. However, the *MVK* and *DXR* transcriptional profiles in model plants were already extensively studied [8,34]. *MVK* was highly expressed in roots and flowers, while the transcript levels of *DXR* was more abundant in photosynthetic tissues [7,8,34].

We verified the expression levels of *CaMVK* and *CaDXR* genes in *C. arabica* organs using RNA-seq data and RT-qPCR. We observed higher transcript levels of these genes in flowers for both analyses. The high levels of expression observed in flowers can be explained by the fact that several terpenes are produced in this organ [34,35], most of which are plant volatile compounds, such as limonene, one of the main monoterpene compounds responsible for the aroma of coffee [22]. In addition, we observed in the RNA-seq data that *CaMVK* and *CaDXR* are upregulated in coffee fruits under MJ treatment compared to mock-control plants, including most of the genes involved in the MVA and MEP pathways. Several studies already described methyl jasmonate function as a potent elicitor of secondary metabolite production [36,37] and hexanoic acid as a plant resistance inductor by producing natural products [24,25].

Our RT-qPCR results showed that hexanoic acid is able to upregulate expression of *CaMVK* in *C. arabica* cv. Obatã roots, a result that is in agreement with previous reports investigating gene expression in *Citrus* plants [25]. In the same Hx experiment, the *DXR* gene was downregulated in the leaves of *C. arabica* cv. Catuaí Vermelho, suggesting that modulation of expression might be genotype-specific. The upregulation of transcription of the *MVK* gene in Hx-treated roots was detected only in the Obatã cultivar. Upregulation of the terpenoid pathway can provide protection against stresses, as previously observed in studies investigating tobacco [38], tomato [39] and maize [40]. In addition, it is important to highlight that Obatã and Catuaí Vermelho cultivars have distinct breeding stories, and they are classified in distinct genetic groups [41]. In this study, we provide insights to show that distinct genetic groups generated by coffee breeding might result in dissimilar transcriptional responses.

## 4. Materials and Methods

### 4.1. Identification and Annotation of MVK and DXR Gene Families in Plants

The coding sequences (CDSs) of *MVK* and *DXR* were retrieved for 24 plant species with sequenced genomes within the PLAZA 4.0 platform [42]. The coding sequences were translated into amino acids using TranslatorX [43]. Gene characterizations were based on characteristics observed in *A. thaliana MVK* and *DXR* genes (AT5G27450 and AT5G62790). Sequences on the PLAZA platform that contained the InterPro domains IPR006205 (Mevalonate kinase) and IPR013750 (GHMP kinase, C-terminal domain) were further selected as *MVK* genes. To identify *DXR*, we retrieved genes that simultaneously contained the InterPro domains IPR003821 (DXP_reductoisomerase), IPR013512 (DXP_reductoisomerase_N), and IPR026877 (DXP C-terminal domain reductoisomerase) for further analysis.

### 4.2. Multiple Sequence Alignments and Phylogenetic Analysis

Coding nucleotide sequences were aligned with MUSCLE [44] and TranslatorX software [43]. In TranslatorX, we were able to eliminate positions containing gaps and missing data with the Gblocks tool [45]. The evolutionary history of genes was inferred using the maximum likelihood method based on the LG model [46]. The consensus tree bootstrap inferred from 1000 replicates was used to represent the evolutionary history of genes analyzed. The percentages of the replicated trees in which the associated rates were grouped in the bootstrap test (1000 repetitions) were shown near the branches. Evolutionary analysis and final trees were performed using MEGAX [47]. To predict the cellular localization of proteins produced by MVK and DXR genes, we used the TargetP 1.1 Server [48], CELLO v.2.5 [49] and Plant-mPLoc [50].

### 4.3. Determination of Gene Structures

To analyze the exon–intron structure of MVK and DXR genes, we used the Gene Structure Display Server v2.0 program with its default parameters [51]. The genomic and CDS in FASTA format corresponding to genes of 24 plant species were inserted to generate gene structures.

### 4.4. Selection Pressure and Evolutionary Analyses

Non-synonymous (dN) and synonymous (dS) nucleotide substitutions within DXR and MVK genomic sequences were used to calculate dN/dS ratios. The indices dN/dS = 1, dN/dS < 1 and dN/dS > 1 represent neutral evolution, purifying selection or positive Darwininan selection, respectively. Individual dN/dS indices for each amino acid of predicted proteins for each gene were also determined using the statistical test suite available on the Selecton Server platform (http://selecton.tau.ac.il/) [52], using the following models: M8 (ωs ≥ 1), M7 (beta) and M5 (gamma). In addition, FEL, SLAC and MEME methods with default settings incorporated into the Datamonkey web interface [53] were also used to identify selection type.

### 4.5. Gene Duplication Analysis

The alignment of duplicate gene pairs of *MVK* and *DXR* in *P. trichocarpa, G. raimondii, R. communis, M. acuminata, S. italica, C. quinoa* and *G. max* species was performed using MUSCLE program implemented in MEGAX [47]. Then, pairwise synonymous (dS) and non-synonymous (dN) numbers of substitutions were calculated using MEGAX. Based on synonymous substitutions per year (λ) equal to 6.5 × 10^−9^ for monocotyledons [54] and 1.5 × 10^−8^ for dicotyledons [55], by substituting the calculated dS values, the approximate age of the duplicated events of the duplicate *DXR* or *MVK* gene pairs was estimated using the following Equation (1):

T  =  Ks/2λ × 10^−6^ mya. (1)

Synteny information was retrieved from the PLAZA 4.0 platform [42].

### 4.6. Gene Recombination Analysis

Recombination events between divergent nucleotide sequences were investigated to detect recombination signals using RDP.v4 software with default parameters (http://web.cbio.uct.ac.za/~darren/rdp.html). In this study, we used the following methods: RDP [56], Geneconv [57] and MaxChi [58] and the *p*-value was set to 0.05.

### 4.7. RNA-seq-Based Expression Profile Analysis of MVK and DXR Genes

We analyzed the digital expression profile of *A. thaliana*, *C. canephora*, *P. trichocarpa*, *O. stiva*, *S. bicolor* and *S. lycopersicum* genes in leaf, root, fruit and flower tissues from the Gene Expression Atlas platform (http://www.ebi.ac.uk/gxa) [59]. *C. arabica* digital expression data from leaves, flowers, fruits and fruits treated with methyl jasmonate (2 mM) were obtained from previously published work [28]. Figures depicting expression maps were developed using Genesis software [60].

### 4.8. Transcriptional Profiles of MVK and DXR Genes in C. arabica by RT-qPCR

#### 4.8.1. Plant Material

Samples were obtained from 20-year-old individual *C. arabica* cv. IAPAR59 plants grown at the Agronomic Institute of Paraná (Londrina—Brazil) under full-sun field conditions with standard irrigation and fertilization practices. We collected open flowers and fruits 120 DAF in January of 2013. Fruit tissues were separated into pulp, perisperm and endosperm, and only perisperm and endosperm tissues were selected for RT-qPCR analysis. All samples were collected between 9 and 11 a.m., transferred immediately to liquid nitrogen and stored at -80°C until RNA was extracted.

#### 4.8.2. Hexanoic Acid Treatment: Experimental Procedures

Five-month-old plants of *C. arabica* cv. Catuaí Vermelho IAC 144 and Obatã IAC 1669-20 (4–5 leaf pairs) were used to assess treatment with hexanoic acid. Plants were selected based on size uniformity, and were transferred to dark pots containing 3 L of aerated nutrient solution (ANS), adapted from Clark (1975) by de Carvalho et al. (2013) [61,62]. The nutrition solution was composed of K_2_SO_4_ (1068 mM), MgSO_4_·7H_2_O (332.5 mM), KH_2_PO_4_ (266 mM), CaCl_2·_2H_2_O (66 mM) and NH_4_NO_3_ (5333 mM). Iron and micronutrients were supplied by using a commercial chelated salt mixture (ConMicros Standard, Conplant) at the following concentrations (in µg.L^−1^): Fe (363), Cu (91), Zn (37), Mn (91), B (91), Mo (18) and Ni (17). Experiments were carried out in a plant growth room, with the temperature set to 23 °C (ranging between 21 °C and 25 °C). Plants were maintained using a 12 h day/night cycle. LED panels with a photosynthetically active photon flux density of approximately 400 μmol.m^−2^.s^−1^ provided artificial lighting. The pH of the nutrient solution was adjusted and maintained between 5.5 and 5.6 daily using chloridric acid or sodium hydroxide. Plants were acclimatized approximately 96 h. After the acclimatization period, nutrient solutions were replaced. The following treatments were assessed: (a) ANS (control); (b) ANS + hexanoic acid (Merck, final concentration 0.55 mM) for 48 h. Plants were grown in 3 to 6 plastic pots in which three plants received each treatment. The potted plants were grouped in “pools” (made of 9 to 18 plants), which were considered a replicate. The experiments were repeated 3 times to obtain biological replicates. The mature leaves of the middle third and lateral roots of the plants were collected within the 3rd hour of the light period, macerated in liquid nitrogen and stored in a freezer at −80°C until RNA extraction was performed.

#### 4.8.3. Transcriptional Profiles of MVK and DXR Genes using RT-qPCR and Statistical Analyses

Total RNA from *C. arabica* mature leaves, roots, flowers and fruits were extracted using the RNeasy Plant kit (Qiagen, Hilden, North Rhine-Westphalia, Germany). Total RNA samples were purified using the RNeasy Minielute Cleanup kit (Qiagen, Hilden, North Rhine-Westphalia, Germany). RNA integrities were verified via 1% agarose gel electrophoresis. The purity of RNA was determined using a NanoDrop ND-100 spectrophotometer (Thermo Scientific, San Jose, USA) and concentrations were obtained using Qubit fluorimeter (Thermo Fisher Scientific, Wilmington, DE, USA). Complementary DNAs (cDNAs) were synthesized using the High Capacity cDNA Reverse Transcriptase kit (Thermo Fisher Scientific, Wilmington, DE, USA) according to the manufacturer’s instructions. A final volume of 20 μL with 5 μg of total RNA was used. Primers were designed with Primer 3 software to amplify products ranging from 95 to 127 bp (Table S1). Primer efficiencies were calculated using LinRegPCR software v. 11.0 [63]. RT-qPCR reactions were performed using a QuantiStudio3 system (Applied Biosystems, Carlsbad, CA, USA) in accordance with basic procedures reported in a previous publication in coffee plants [28]. The reaction mixture contained 7.5 μL of GoTaq^®^ Hot Start Polymerase (Promega, Madison, WI, USA), 0.3 μL of each primer (3 μM), 1 μL of cDNA (10 ng/μL) and 5.5 μL of Milli-Q water in a final volume of 15 μL. The RT-qPCR conditions began with an initiation step at 95 °C for 2 min; and were followed by 40 cycles at 94 °C for 30 s and 60 °C for 60 s. Melting curves were assessed to verify the presence of a single product and included a negative control. All reactions were performed with three biological and technical replicates, and we followed the MIQE guidelines for RT-qPCR experiments [64].

Relative expression was calculated using the following formula (Equation (2)):

(1 + E) ^−∆∆Ct^ (2)

where E = primer efficiency, ΔCt = Ct _target gene_–Ct _reference gene_ and ΔΔCt = ΔCt _target_–ΔCt _internal calibrator_, as previously described [62]. In all cases, *GAPDH* was the normalizer, as recommended for coffee plants [62,65]. Internal calibrators were endosperm for IAPAR59 and control samples (without application of hexanoic acid) for the hexanoic acid experiment. Data were analyzed via two-way ANOVA and the post-hoc Tukey test (*p* < 0.05) using XLSTAT.2014 software [66].

## 5. Conclusions

No *MVK* genes were identified in *C. reinhardtii* in the present study, suggesting a very specific evolution pattern for terpenoid biosynthesis in Chlorophyta. Seven species have more than one copy of the *MVK* gene, and six species had more than one copy of *DXR*. Most duplications are due to WGD and also happened with other genes of the pathway, except for *R. communis* and *S. italica*. The *DXR* gene was subjected to a stronger purifying selection pressure, producing lower dN/dS values compared to *MVK*. Our analyses still raise the need for further studies characterizing the impact that *MVK* knockdown could have on the phenotype and terpenoid production of model plants.

Furthermore, the *DXR* gene was shown to undergo a greater number of recombinations. We observed that *DXR* is more transcriptionally active than *MVK* in leaves, while *MVK* transcripts are more abundant in angiosperm roots and flowers. We finally observed that Hx is able to modulate the expression of these genes in coffee leaves and roots in a genotype-specific manner.

## Figures and Tables

**Table 1:** Identification of *MVK* and *DXR* genes in 24 plant species.

Lineage	Species	Gene ID	
		*MVK*	*DXR*
**Outgroups**	*Amborella trichopoda*	*ATR0561G047*	*ATR0730G147*
	*Chlamydomonas reinhardtii*		*Cre12.g546050*
	*Marchantia polymorpha*	*Mapoly0214s0007*	*Mapoly0064s0074*
			*Mapoly0034s0078*
**Eudicotyledons**	*Arabidopsis thaliana*	*AT5G27450*	*AT5G62790*
	*Capsella rubella*	*Carubv10001182m.g*	*Carubv10026322m.g*
	*Capsicum annuum*	*CAN.G1077.21*	*CAN.G781.11*
	*Chenopodium quinoa*	*AUR62005132*	*AUR62030965*
		*AUR62000841*	*AUR62008212*
	*Coffea arabica*	*CaMVK_comp31934*	*CaDXR_comp33448*
	*Coffea canephora*	*Cc00_g15600*	*Cc04_g14010*
	*Cucumis sativus*	*Cucsa.091600*	*Cucsa.196910*
	*Eucalyptus grandis*	*Eucgr.B01993*	*Eucgr.B00316*
	*Glycine max*	*Glyma.03G239000*	*Glyma.16G08900*
		*Glyma.19G236200*	*Glyma.17G089600*
			*Glyma.05G037500*
	*Gossypium raimondii*	*Gorai.007G296000*	*Gorai.007G001700*
		*Gorai.007G342800*	*Gorai.006G189200*
	*Populus trichocarpa*	*Potri.013G024000*	*Potri.012G080900*
		*Potri.005G034600*	*Potri.015G076200*
		*Potri.005G035300*	
	*Prunus persica*	*Prupe.6G327600*	*Prupe.5G174000*
	*Ricinus communis*	*RCO.g.30128.000221*	*RCO.g.30147.000220*
		*RCO.g.29656.000023*	
	*Solanum lycopersicum*	*Solyc01g098840.2*	*Solyc03g114340.2*
	*Theobroma cacao*	*TCA.TCM_026574*	*TCA.TCM_01200*
	*Utricularia gibba*	*UGI.Scf00073.6483*	*UGI.Scf00226.11960*
**Monocotyledons**	*Brachypodium distachyon*	*Bradi2g13360*	*Bradi2g00650*
	*Musa acuminata*	*MAC03G2585*	*MAC12G2064*
		*MAC04G2409*	*MAC07G0719*
	*Oryza sativa*	*Os10g18220*	*Os01g01710*
	*Setaria italica*	*Seita.3G273700*	*Seita.5G071800*
		*Seita.2G354500*	
	*Sorghum bicolor*	*Sobic.004G012400*	*Sobic.003G103300*

**Table 2:** Average non-synonymous (dN) and synonymous (dS) substitution ratios for *MVK* and *DXR* genes in eudicotyledonous and monocotyledonous plants.

Gene		dN/dS		Positive Sites *		Selection	
***MVK***	**Models**	**Eudycots**	**Monocots**	**Eudycots**	**Monocots**	**Eudycots**	**Monocots**
	**M8(beta+w>=1)**	0.19831	0.29343	112, 206, 213	0	Purifying	Purifying
	**M7(beta)**	0.22751	0.35528	0	0	Purifying	Purifying
	**M5(gamma)**	0.18986	0.26649	0	0	Purifying	Purifying
***DXR***	**M8(beta+w>=1)**	0.11136	0.14043	0	0	Purifying	Purifying
	**M7(beta)**	0.11877	0.14589	0	0	Purifying	Purifying
	**M5(gamma)**	0.16233	0.13052	0	0	Purifying	Purifying

* Calculations were performed using a Bayesian inference approach in Selecton Server (*p* < 0.05) using three distinct selection models.

**Table 3:** Predicted number and location of codons under positive selection in *MVK* and *DXR.*

Gene		Positive Selection Sites *		Selection	
***MVK***	**Methods**	**Eudicot.**	**Monocot.**	**Eudycot.**	**Monocot.**
	**FEL**	0	0	Purifying	Purifying
	**SLAC**	0	0	Purifying	Purifying
	**MEME**	42, 129, 330	42, 115, 122, 317	Purifying	Purifying
***DXR***	**FEL**	0	0	Purifying	Purifying
	**SLAC**	0	0	Purifying	Purifying
	**MEME**	12, 48, 52, 332, 409	142, 145	Purifying	Purifying

* Calculations were performed using the Datamonkey platform (*p* < 0.05).

**Table 4:** Duplication time estimated by dN/dS analysis in *MVK* genes of seven plant species.

Species	Duplicated *MVK* Genes		dN	dS	dN/dS	Time (mya)
*Populus trichocarpa*	*Potri.005G034600*	*Potri.005G035300*	0.02	0.02	1.00	0.67
	*Potri.005G034600*	*Potri.013G024000*	0.05	0.20	0.25	6.67
	*Potri.005G035300*	*Potri.013G024000*	0.04	0.19	0.21	6.33
*Gossypium raimondii*	*Gorai.007G296000*	*Gorai.007G342800*	0.11	0.46	0.24	15.33
*Ricinus communis*	*RCO.g.30128.000221*	*RCO.g.29656.000023*	0.06	0.27	0.22	9.00
*Musa acuminata*	*MAC03G2585*	*MAC04G2409*	0.10	0.46	0.22	35.38
*Setaria italica*	*Seita.3G273700*	*Seita.2G354500*	0.02	0.06	0.33	4.62
*Chenopodium quinoa*	*AUR62005132*	*AUR62000841*	0.02	0.08	0.25	2.67
*Glycine max*	*Glyma.03G239000*	*Glyma.19G236200*	0.03	0.17	0.18	5.67

Abbreviation: mya, million years ago.

**Table 5:** Duplication time estimated by dN/dS analysis for *DXR* genes in six plant species.

Species	Duplicated *DXR* Genes		dN	dS	dN/dS	Time (mya)
*Gossypium raimondii*	*Gorai.007G001700*	*Gorai.006G189200*	0.06	0.34	0.18	11.33
*Marcanthia polymorpha*	*Mapoly0064s0074*	*Mapoly0034s0078*	0.15	n/c	-	-
*Populus trichocarpa*	*Potri.012G080900*	*Potri.015G076200*	0.02	0.16	0.13	5.33
*Musa acuminata*	*MAC12G2064*	*MAC07G0719*	0.05	0.46	0.11	15.33
*Chenopodium quinoa*	*AUR62030965*	*AUR62008212*	0.04	0.09	0.44	3.00
*Glycine max*	*Glyma.16G08900*	*Glyma.17G089600*	0.14	0.6	0.23	20.00
	*Glyma.16G08900*	*Glyma.05G037500*	0.15	0.58	0.26	19.33
	*Glyma.17G089600*	*Glyma.05G037500*	0.02	0.07	0.29	2.33

Abbreviations: mya, million years ago; n/c, not calculated.

**Table 6:** Recombination events predicted for the *MVK* gene family using the RDP, GENECONV and MAXCHI recombination methods.

Species	RDP	GENECONV	MAXCHI	Gene ID
*E. grandis*	1	0	4	*Eucgr.B01993*
*C. rubella*	0	0	2	*Carubv10001182m.g*
*M. polymorpha*	0	0	3	*Mapoly0214s0007*
*M. acuminata*	0	0	1	*MAC04G2409*
*S. italica*	0	0	5	*Seita.3G273700*

**Table 7:** Recombination events predicted for the *DXR* gene family using the RDP, GENECONV and MAXCHI recombination methods.

Species	RDP	GENECONV	MAXCHI	Gene ID
*C. sativus*	2	0	0	*Cucsa.196910*
*T. cacao*	0	1	1	*TCA.TCM_01200*
*G. raimondii*	0	3	0	*Gorai.007G001700*
*R. communis*	0	0	2	*RC30147G06220*
*C. annuum*	0	0	5	*CANG781.11*
*S. bicolor*	0	0	3	*Sobic.003G103300*
*M. acuminata*	0	0	4	*MAC07G0719*
*M. polymorpha*	0	0	6	*Mapoly0064s0074*

## Supplementary Materials

The following are available online at https://www.mdpi.com/2223-7747/9/4/525/s1. Figure S1. Schematic representation of genomic regions containing *MVK* or *DXR* genes; Figure S2. RPKM values of *C. arabica* terpenoid pathway genes under methyl jasmonate application; Figure S3. RPKM values of *C. arabica* terpenoid pathway genes in flowers, leaves and along fruit development; Figure S4. Distribution of dN/dS over individual sites in *MVK*, based on Selecton statistics; Figure S5. Distribution of dN/dS over individual sites in *DXR*, based on Selecton statistics; Table S1. Primer sequences used for RT-qPCR analyses**.**
