# Supplementary material for: Low-Copy Genes in Terpenoid Metabolism: The Evolution and Expression of MVK and DXR Genes in Angiosperms
Source: Plants (Basel). 2020 Apr 19;9(4):525. doi: 10.3390/plants9040525 (PMC7238024; doi:10.3390/plants9040525)
Supplement: Supplementary file 1 [file plants-09-00525-s001.pdf]

# Supplementary Materials

**Supplementary Table 1**. Primer sequences used for RT-qPCR analyses

| Gene ID | Primer sequence | Size (bases) | Tm (°C) | Amplicon (bp) | Efficiency |
|---|---|---|---|---|---|
| *CaMVK* | F: ATGTTTCCCTCCGCTTCC | 18 | 59,3 | 95 | 97% |
| | R: TCCAACCGGCCAAGAAAAT | 19 | 59,1 | | |
| *CaDXR* | F: CCTGTTCTCCCCTACGACT | 19 | 59,3 | 127 | 92% |
| | R: GCCTCCATTGTCCTTCCT | 18 | 59,1 | | |
| *CaGAPDH* | F: AGGCTGTTGGGAAAGTTCTTC | 21 | 59,5 | 70 | 98% |
| | R: ACTGTTGGAACTCGGAATGC | 20 | 58.4 | | |

## Supplementary Figures

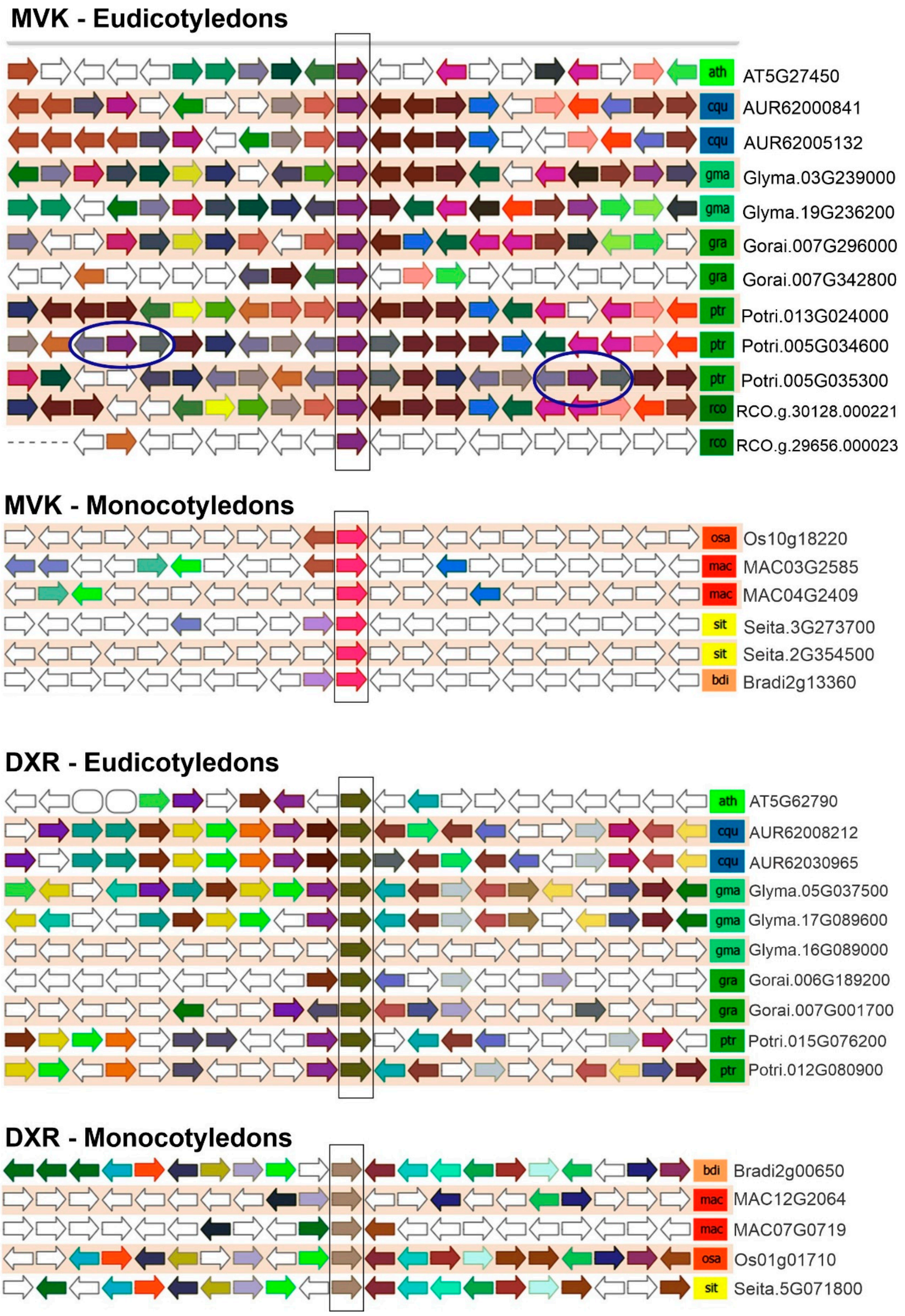


**Supplementary Figure 1.** Schematic representation of genomic regions containing *MVK* or *DXR* genes. We highlighted in a rectangle *DXR* and *MVK*. The figure was based on the "Synteny plot" tool of Plaza 4.0. Genes with the same color have similar function. Eudicot *MVK* genes share

similar genomic neighborhood. We circled the *MVK* tandem duplication in *P. trichocarpa*. In monocots, genomic neighborhood among MVK genes has less collinear genes. In *DXR*, we also observe high similar genomic neighborhood, suggesting WGD events in soybean (Glyma) and *P. trichocarpa* (Potri). Only one gene in soybean (Glyma.16G089000) and one in cotton (Gorai.006G189200) do not share a significative genomic neighborhood, suggesting that these genes arose from segmental duplication.

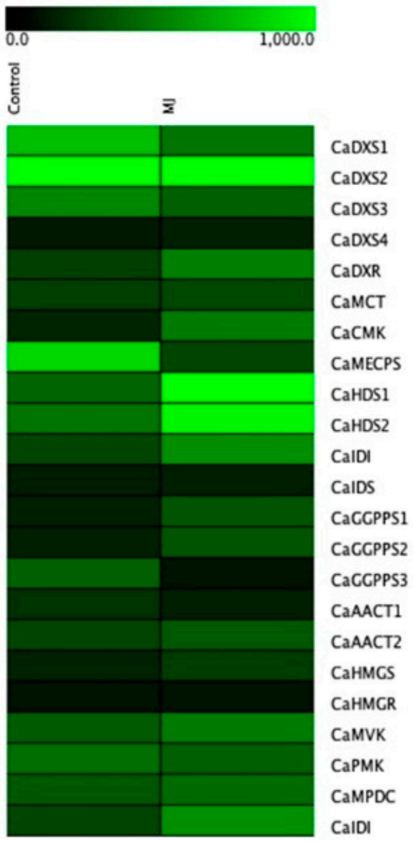


**Supplementary Figure 2.** RPKM values of *C. arabica* terpenoid pathway genes under methyl jasmonate application. Legend: C – control; MJ – methyl jasmonate application.

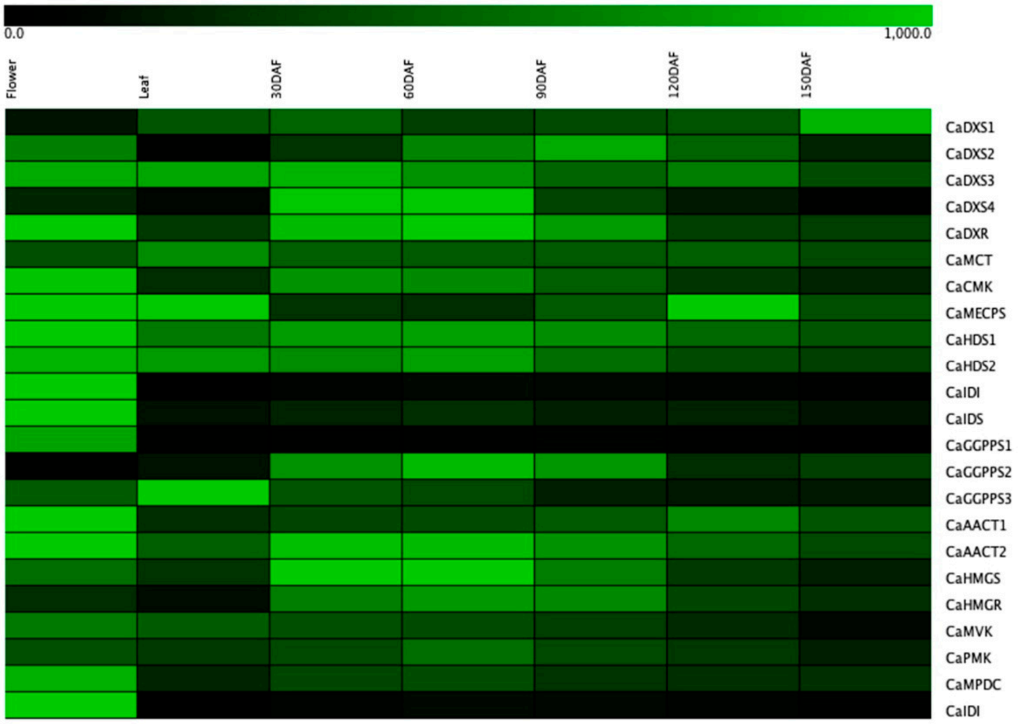


**Supplementary Figure 3.** RPKM values of *C. arabica* terpenoid pathway genes in flowers, leaves and along fruit development. Legend: DAF – perisperm in days after flowering.

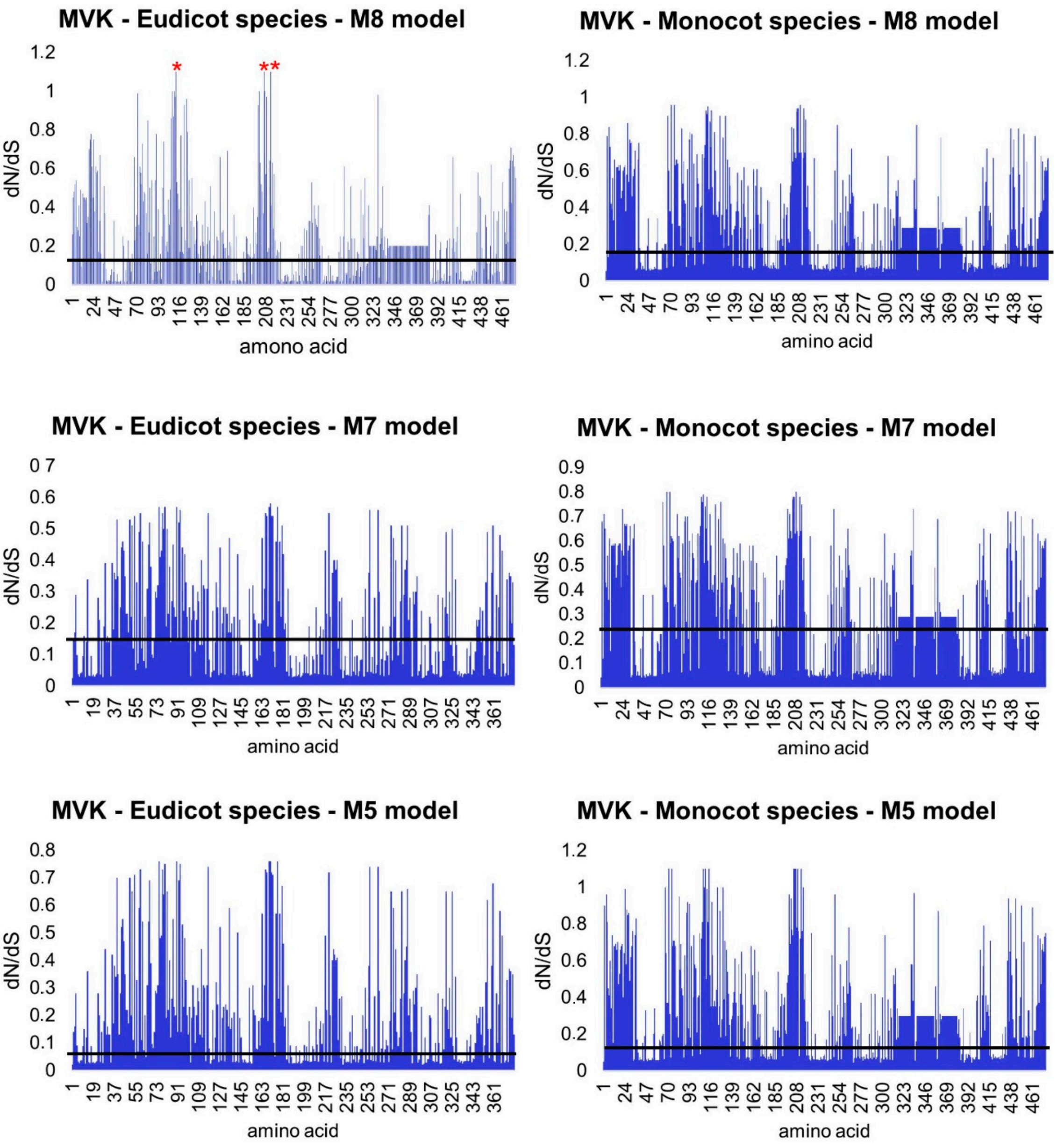


**Supplementary Figure 4.** Distribution of dN/dS over individual sites in *MVK*, based on Selecton statistics. The horizontal line represents the average dN/dS. Asterisks (*) represent statistical significance in Bayesian analysis ($p < 0,05$).

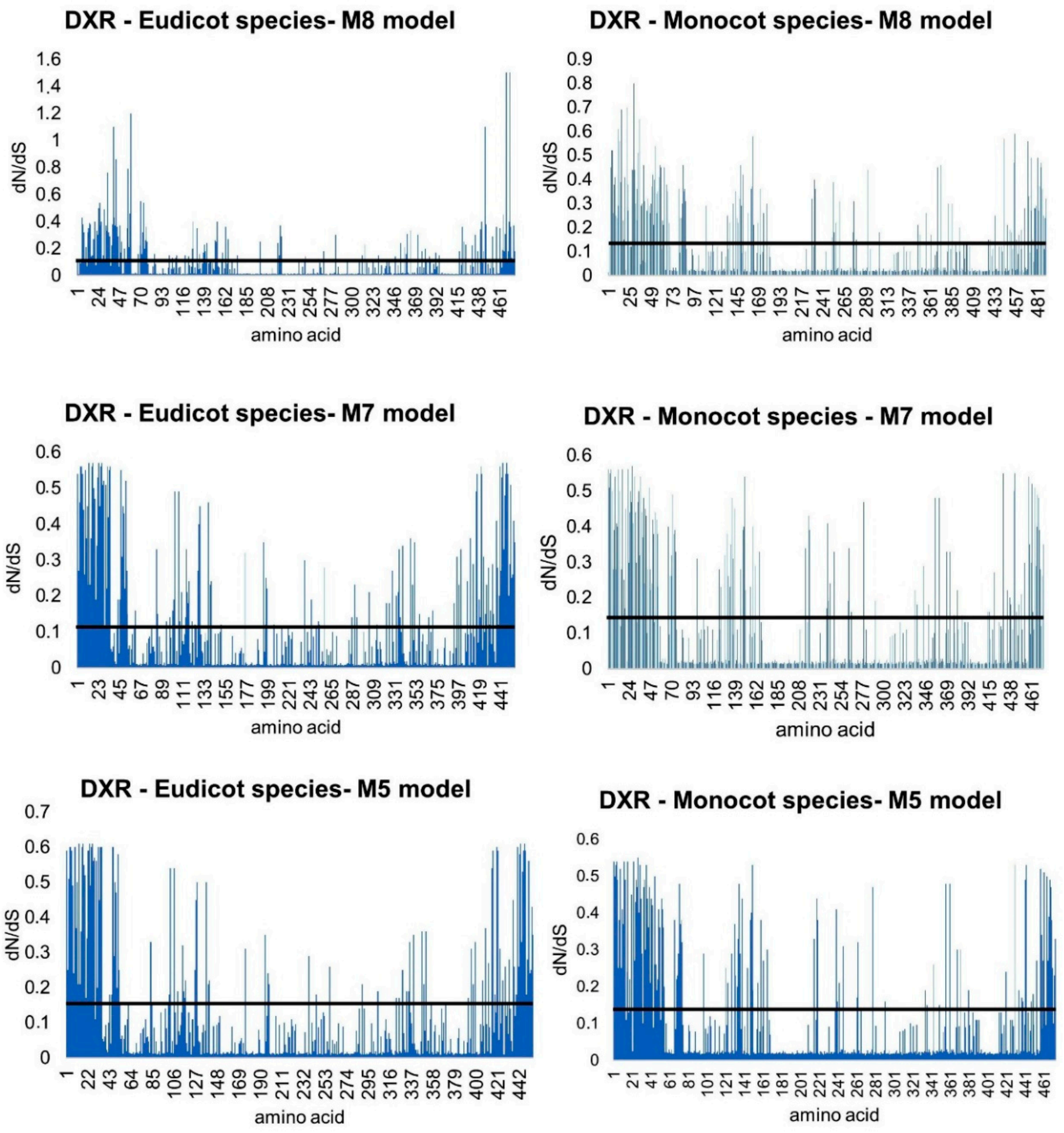


**Supplementary Figure 5.** Distribution of dN/dS over individual sites in *DXR*, based on Selecton statistics. The horizontal line represents the average dN/dS.
